# Satisfaction with life and the risk of occupational injury

**DOI:** 10.1186/s40557-018-0260-x

**Published:** 2018-08-02

**Authors:** Sung-Min Park, Hwan-Cheol Kim, Shin-Goo Park, Hyun-Suk Jang, Go Choi, Jong-Han Leem

**Affiliations:** 1 Department of Occupational and Environmental Medicine, School of Medicine, Inha University Hospital, 7-206 3rd St. Shinhung-dong, Jung-gu, Incheon, 400-711 Republic of Korea; 2 Department of Social and Preventive Medicine, School of Medicine, Inha University, Incheon, Republic of Korea; 3 Department of Occupational and Environmental Medicine, School of Medicine, Inha University, Incheon, Republic of Korea

**Keywords:** Satisfaction with life, Occupational injury, Occupational accident

## Abstract

**Background:**

Occupational injuries increase burden on society as well as personal health. Low satisfaction with life may not only increases the risk of occupational injuries directly, but also influences other factors that increase the risk of occupational injury. Along with previous studies on the risk of occupational injury, we sought to explore the relationship between satisfaction with life and occupational injury.

**Methods:**

The study participants were 6234workers health screened at a university hospital in Incheon. Information on occupational injury and satisfaction with life scale (SWLS) was obtained in a self-report format. Participants were allocated to one of four SWLS groups; the dissatisfied group, the slightly dissatisfied group, the slightly satisfied group, and the satisfied group. The analysis was performed using the chi-square test primarily and by logistic regression adjusted for potential confounders.

**Results:**

In men, the un-adjusted and adjusted odds ratios (ORs) of low satisfaction with life (SWLS< 20) were 1.98(CI1.55–2.53) and 1.81(CI 1.41–2.32), respectively. When the SWLS were divided into four groups, the adjusted ORs of the slightly satisfied (20–25), slightly dissatisfied(15–19), and dissatisfied(≤14) groups were 1.21, 1.72, and 2.70, respectively. That is ORs tended to increase linearly with decreasing SWLS score (p for trend < 0.001). In women, this relation was of borderline significance at best.

When subjects were dichotomized based on SWLS scores, for males, the cured and adjusted RRs of occupational injury in the low satisfaction with life group were1.91 (95% CI: 1.50–2.42) and 1.66 (95% CI: 1.30–2.13), and for females, the adjusted-RR was marginally significant (1.67; 95% CI: 0.93–2.99).

When subjects were divided into four groups by SWLS scores, adjusted RRs tended to increase linearly with decreasing SWLS score for males (slightly satisfied: 1.18, 95% CI: 0.77–1.82; slightly dissatisfied: 1.65, 95% CI: 1.08–2.52; dissatisfied: 2.22, 95% CI: 1.44–3.42; p for trend < 0.001) and for females (slightly satisfied: 1.17, 95% CI: 0.42–3.30; slightly dissatisfied: 1.56, 95% CI: 0.56–4.36; dissatisfied: 2.38, 95% CI: 0.84–6.74; p for trend = 0.040).

**Conclusions:**

This study suggests that the risk of occupational injury was higher in workers not satisfied with life, and indicates attention to satisfaction with life may promote the health of workers.

## Background

Occupational injury refers to bodily damage caused by work that exposes the individual to harmful factors. Hands, feet, vertebrae, skeleton, skin are often injured. These harmful factors may be physical, chemical, biological or psychological and involve temperature, noise, insect pests, chemicals, radiation, and others [1]. More than 350,000 people die at work annually worldwide and more than 270,000,000 are injured while working [2]. According to an analysis of the status of industrial accidents issued by the Korean Ministry of Employment and Labor, the overall accident rate in 2017 was 0.48 per 100 persons, which was 2.0% down on the previous year, but the number of deaths and occupational diseases increased by 10.1 and 16.6%, respectively, from the previous year. Because major industries differ between country and risks of exposure vary by industry, incidences of occupational injuries differ widely, though they generally high in agricultural, fishery, and forestry sectors [3]. In developed countries, rates of injuries to the spine, hands and wrists are high in the construction industry [4] and manufacturing [5]. According to a report issued by the National Institute for Occupational Safety and Health (NIOSH) in the USA, 15 workers die per day from traumatic injuries in the United States and 200 workers are hospitalized [6].

These occupational injuries not only undermine worker health, they also reduce productivity and increase social burdens. Therefore, it is important that we identify factors that increase the risk of occupational injury and manage them to reduce the rate of occupational injuries. According to previous studies, the most important personal risk factor of occupational injury is age. For example, older workers tend to suffer from back pain at work, whereas younger workers do not [4]. In addition to this, obesity [7, 8] education and training [9, 10], high-performance work systems that involve extensive training, variety, and autonomy [11], and quality of life(QOL) are known to be associated with occupational injury [12].

The Satisfaction With Life Scale (SWLS) [13], which was introduced in 1985, has been widely used to measure life satisfaction components of subjective well-being. It is known that mental health as measured by the SWLS can be used as an indicator of future behaviors, such as, suicide [14], and that mental health disorders directly influence worker health and absenteeism [15]. On the other hand, satisfaction with life is thought to be related to psychological factors (i.e., depression, stress, and anxiety). Indirect effects mediated by adverse health behaviors and inadequate safety communications may affect the risk of occupational injury in workers with lower satisfaction with life. Accordingly, we considered that satisfaction with life might be associated with the occurrence of occupational injuries. Furthermore, because satisfaction with life is commonly used as a personal indicator, we considered identifying the relationship between this indicator and risk of occupational injury might provide a means to effectively reducing the risk of occupational injury.

However, cross-sectional designs and small populations make it difficult to determine the impact of satisfaction with life on occupational injury. Furthermore, due to the potential for occupational injury to impact satisfaction with life, subject recall of situations in which occupational injuries occurred may be distorted. Therefore, we conducted this prospective study on workers in various industrial sectors. The aim of this study was to determine whether satisfaction with life influences the risk of occupational injury.

## Methods

### Study population

The study participants had varied employments in accommodation, manufacturing, aviation, and others, and all underwent a health examination at a university hospital in Incheon in accord with the requirements of the Korean Industrial Safety and Health Act. In 2012, 10,482 workers were invited to fill out a self-reporting questionnaire that addressed personal and occupational characteristics, and occupational injury, and included the SWLS. A second survey was conducted on workers from the same business entities from 2013 to 2015, during which workers were asked to complete to a self-reporting questionnaire that addressed occupational injury. A total of 7071 (67.4%) workers responded to the second survey, but because of incomplete responses and missing data on absence from work, 837 subjects were excluded. Thus, the analysis was conducted on 6234(59.4%) subjects (4610 males and 1624 females). The study protocol was approved by the institutional review board of Inha University Hospital. All responders provided informed written consent.

### Satisfaction with life

Satisfaction with life were measured using the Satisfaction With Life Scale(SWLS), which was developed by Ed Diener and colleagues [13] to assess the cognitive component of subjective well-being and is probably the most well-used scale in scientific studies on life satisfaction. Previous studies have shown the SWLS has satisfactory inter-item correlations, reliability and validity [16]. The SWLS consists of 5 items that relate to global satisfaction with life; (1) In most ways my life is close to my ideal, (2) The conditions of my life are excellent, (3) I am satisfied with my life, (4) I have achieved the important things I wanted, and (5) If I could live my life over, I wouldn’t change anything. Each item is scored on a scale from one to seven points, giving a total score range of 5–35 points, where a high score indicates satisfaction with life. We interpreted SWLS scores of ≥20 to indicate satisfaction with life and low scores to indicate dissatisfaction. In addition, SWLS scores were divided into quartile groups to determine the nature to the association between SWLS scores and occupational injury, as follows: the dissatisfied group (SWLS score ≤ 14), the slightly dissatisfied group (score 15–19), the slightly satisfied group (score 20–25), and the satisfied group (score ≥ 26).

### Covariates

Possible confounding factors that might be associated with occupational injury, as indicated by previous studies were included in the analysis [17–20]. These potential confounders included; self-reported age (years; categorized as < 30, 30–39, 40–49, 50–59, or ≥ 60), gender(male and female), marital status(never married, married, divorced/widowed), education status(middle school, high school, college), chronic disease(yes or no), smoking status (never, former, or current), and alcohol consumption, and occupational characteristics, such as, industry classification, employment status(regular, temporary), type of work (shift or non-shift), job tenure (< 1 year, or 1–4, 5–9, or ≥ 10 years), and working hours per week(< 40, 41–59, ≥60).

### Occupational injury

Occupational injury histories were determined using questionnaire responses. Those that responded “yes” to either (1) “During the last 12 months were you ever hospitalized because of a work-related accident?” or (2) “During the past 12 months were you absent from work for more than 1 day due to a work-related accident?” constituted the occupational injury group.

### Statistical analysis

We analyzed the data of men and women separately because gender can influence occupational injury rates and satisfaction with life. Experiences of occupational injury according to the participant’s general and occupational characteristics and life satisfaction scale were analyzed using the Chi-squared test. In order to calculate the risk ratio (RR) of satisfaction with life for occupational injury, we constructed a binomial generalized linear model (GLM) with a log link.

function. Adjusted RRs were calculated after adjusting for potential confounders. Age, educational level, smoking status, industry, employment status, tenure, and working hours were adjusted for males, while age, marital status, chronic disease, industry, shift work, tenure, and working hours were adjusted for females. These confounding variables were selected based on results of Chi-squared testing by gender. Data were analyzed using SPSS v.19.0 software (SPSS Inc., Chicago, IL, USA).

## Results

General characteristics and occupational injuries are summarized in Table 1. The risk of occupational injury was higher among male workers (6.4%) than among female ones (3.5%). In females, the risk of occupational injury was highest in the fifties (6.4%), and lowest in the thirties (1.8%). In males, the risk of occupational injury among high school graduates was 7.4%, and this was significantly higher than those of middle school graduates and college graduates (4.6 and 5.0%, respectively), and in terms of smoking status, it was highest in current-smokers (7.7%) and lowest in former-smokers (4.7%).For females, those with chronic disease (7.0%) experienced occupational injury than those without (3.2%). Besides, marital status and alcohol consumption were not in a statistically significant correlation with occupational injury in the both males and females.

**Table 1:** General characteristics of men and women that experienced an occupational injury

	Male				Female			
	N	Cases of the occupational injury		*p*-value^a^	N	Cases of the occupational injury		*p*-value^a^
		n	%			n	%	
Total	4610	297	6.4		1624	57	3.5	
Age (years)								
< 30	297	21	7.1	0.433	476	15	3.2	0.007
30–39	1628	112	6.9		570	10	1.8	
40–49	1632	101	6.2		371	19	5.1	
50–59	936	52	5.6		188	12	6.4	
≥ 60	117	11	9.4		19	1	5.3	
Marital status								
Never married	1182	74	6.3	0.899	789	21	2.7	0.133
Married	3356	218	6.5		808	36	4.5	
Divorced/widowed	72	5	6.9		27	0	0.0	
Educational status								
≤ Middle school	195	9	4.6	0.004	107	4	3.7	0.428
High school	2729	203	7.4		1011	31	3.1	
≥ College	1686	85	5.0		506	22	4.3	
Chronic disease								
No	3559	221	6.2	0.236	1482	47	3.2	0.028
Yes	1051	76	7.2		142	10	7.0	
Smoking habit								
Never	1076	63	5.9	0.001	1570	53	3.4	0.159
Former	1299	61	4.7		28	2	7.1	
Current	2235	173	7.7		26	2	7.7	
Alcohol consumption (unit/week)								
0	1119	69	6.2	0.900	979	30	3.1	0.428
1~ 14	2252	146	6.5		567	24	4.2	
15+	1239	82	6.6		78	3	3.8	

^a^Obtained by a Chi-squared test or Fisher’s exact test

In males, significant differences were observed for industrial classification (11.1% for business facilities management and business support services; 8.7% for manufacturing; 8.3% for transportation; 7.4% for wholesale and retail trade; 7.1% for public administration and defense; and so on). Among the females, the risk of occupational injury was tended to increase with job tenure (i.e., 2.5% in those with job tenure of less than a year; 2.4% in those with 1–4 years; 3.5% in those with 5–9 years; and 5.4% in those with 10 years or more), although the difference was only marginally significant (*p* = 0.060). No significant difference according to job tenure emerged with regard to male workers. Among the males, the risk of occupational injury was tended to increase with a working time (i.e., 4.7% in those that worked for < 40 h per week; 6.4% for 41-59 h; 10.4% for ≥60 h). On the other hand, the risk of occupational injury was highest in those that worked for ≥60 h (9.3%), and lowest in those for 41–59 h (2.4%).However, no significant difference in occupational injury experiences was observed in male and female workers according to employment status, and shift work (Table 2).

**Table 2:** Occupational characteristics of men and women that experienced an occupational injury

	Male				Female			
	N	Cases of the occupational injury		*p*-value^a^	N	Cases of the occupational injury		*p*-value^a^
		n	%			n	%	
Industry								
Manufacturing	1374	119	8.7	0.001	526	23	4.4	0.641
Wholesale and retail trade	992	73	7.4		309	10	3.2	
Membership organizations, repair and other personal services	882	25	2.8		28	0	0.0	
Transportation	671	56	8.3		26	0	0.0	
Human health and social work activities	139	2	1.4		594	18	3.0	
Information and communications	138	0	0.0		26	0	0.0	
Electricity, gas, steam and water supply	79	3	3.8		2	0	0.0	
Education	53	0	0.0		19	2	10.5	
Public administration and defense	42	3	7.1		2	0	0.0	
Business facilities management and business support services	27	3	11.1		3	0	0.0	
Sewerage, waste management, materials recovery and remediation activities	22	0	0.0		30	1	3.3	
Professional, scientific and technical activities	22	1	4.5		1	0	0.0	
Others	169	12	7.1		58	3	5.2	
Employment status								
Regular	4135	259	6.3	0.144	1284	46	3.6	0.757
Temporary	475	38	8.0		340	11	3.2	
Shift work								
No	2942	186	6.3	0.659	793	33	4.2	0.163
Yes	1668	111	6.7		831	24	2.9	
Job tenure (years)								
< 1	374	28	7.5	0.178	442	11	2.5	0.060
1–4	576	42	7.3		340	8	2.4	
5–9	987	73	7.4		398	14	3.5	
≥ 10	2673	154	5.8		444	24	5.4	
Hours/week worked								
≤ 40	1837	86	4.7	< 0.001	732	29	4.0	0.003
41–59	1959	126	6.4		795	19	2.4	
≥ 60	814	85	10.4		97	9	9.3	

^a^Obtained by a Chi-squared test or Fisher’s exact test

Table 3 shows the risk of occupational injury according to satisfaction with life scale (SWLS) and gender. The risk of occupational injury in low satisfaction with life group (SWLS score < 20) was significantly higher than those in high satisfaction group (8.6% vs. 4.5%) in males. In females, the risk of occupational injury in the low satisfaction group was greater than in the high satisfaction group (4.3% vs. 2.6%), but the difference was borderline significant (*p* = 0.054).Analysis of quartile groups showed that in males, the risk of occupational injury in the dissatisfied group (SWLS score ≤ 14) was 11.0%, which was significantly higher than in the other groups (*p*-value < 0.001). In females, the risk of occupational injury in the dissatisfied group was 5.7%, and this was greater than in the other quartile groups, but of only borderline significance (*p*-value = 0.067).

**Table 3:** Satisfaction with life scale (SWLS) scores of men and women that experienced an occupational injury

	Male				Female			
	N	Cases of the occupational injury		*p*-value^a^	N	Cases of the occupational injury		*p*-value^a^
		n	%			n	%	
Low satisfaction with life								
No (SWLS≥20)	2415	109	4.5	< 0.001	773	20	2.6	0.054
Yes (SWLS< 20)	2195	188	8.6		851	37	4.3	
SWLS score in 4 groups								
Satisfied (≥26)	785	30	3.8	< 0.001	211	5	2.4	0.067
Slightly satisfied (20–25)	1630	79	4.8		562	15	2.7	
Slightly dissatisfied (15–19)	1371	97	7.1		503	17	3.4	
Dissatisfied (≤14)	824	91	11.0		348	20	5.7	

^**a**^Obtained by a Chi-squared test or Fisher’s exact test

Table 4 presents associations between SWLS scores and risk of occupational injury in males and females. Risk ratios (RRs) were calculated by GLM. In all analyses, confounding variables in each model were selected from the results shown in Tables 1 and 2.

**Table 4:** Risk ratios (95% confidence intervals) of occupational injury for men and women with respect to SWLS scores

	Male		Female	
	Unadjusted	Adjusted^a^	Unadjusted	Adjusted^b^
	RR (95% CI)	RR (95% CI)	RR (95% CI)	RR (95% CI)
Low satisfaction with life				
No (SWLS≥20)	1.00	1.00	1.00	1.00
Yes (SWLS< 20)	1.91 (1.50–2.42)	1.66 (1.30–2.13)	1.62 (0.94–2.81)	1.67 (0.93–2.99)
SWLS score in 4 groups				
Satisfied (≥26)	1.00	1.00	1.00	1.00
Slightly satisfied (20–25)	1.28 (0.84–1.97)	1.18 (0.77–1.82)	1.19 (0.43–3.29)	1.17 (0.42–3.30)
Slightly dissatisfied (15–19)	1.91 (1.26–2.90)	1.65 (1.08–2.52)	1.50 (0.55–4.11)	1.56 (0.56–4.36)
Dissatisfied (≤14)	2.83 (1.86–4.31)	2.22 (1.44–3.42)	2.28 (0.85–6.12)	2.38 (0.84–6.74)
p for trend	< 0.001	< 0.001	0.035	0.040
Per 1 point decrease in SWLS	1.07 (1.05–1.09)	1.05 (1.03–1.08)	1.04 (1.00–1.09)	1.04 (0.99–1.10)

^a^Adjusted for age, educational level, smoking habit, industry, employment status, tenure, and working hours

^b^Adjusted for age, marital status, chronic diseases, industry, shift work, tenure, and working hours

When subjects were dichotomized based on SWLS scores (no vs. yes), in the male crude model, the RR of occupational injury in the low satisfaction with life group was 1.91 (95% CI: 1.50–2.42), and this was significant at 1.66(95% CI: 1.30–2.13) after adjusting for confounders. For females, the unadjusted-RR was 1.62 (95% CI: 0.94–2.81) and the adjusted-RR was marginally but significantly larger (1.67; 95% CI: 0.93–2.99).

On the other hand, when subjects were divided into four groups by SWLS scores, unadjusted RRs tended to increase linearly with decreasing SWLS score (slightly satisfied: 1.28, 95% CI: 0.84–1.97; slightly dissatisfied: 1.91, 95% CI: 1.26–2.90; dissatisfied: 2.83, 95% CI: 1.86–4.31; p for trend < 0.001). Adjusted RRs of occupational injury were as follows; 1.18(95%: 0.77–1.82) in the slightly satisfied group, 1.65(95%: 1.08–2.52) in the slightly dissatisfied group, and 2.22 (95%: 1.44–3.42) in the dissatisfied group. However, the linear trend remained significant (P for trend < 0.001). In the analysis of continuous SWLS scores, adjusted RR increased by 1.05(95% CI: 1.03–1.08) per SWLS point.

For females, adjusted RRs tended to increase linearly with decreasing SWLS score (slightly satisfied: 1.17, 95% CI: 0.42–3.30; slightly dissatisfied: 1.56, 95% CI: 0.56–4.36; dissatisfied: 2.38, 95% CI: 0.84–6.74; p for trend = 0.040). Adjusted RR was found to increase 1.04 (95% CI: 0.99–1.10) per 1SWLS point decrease, though this was of borderline significance (*p* = 0.081). (Table 4).

## Discussion

The present study shows satisfaction with life was significantly associated with occupational injury in men. However, in women, SWLS scores were associated with occupational injury with borderline significance. These trends were maintained after adjustment for potential confounders. The reasons for this difference in gender are unclear, but it may be due to the different attitudes of women, and that women responded that they were dissatisfied with their lives more than men [21–23]. The healthy worker effect raises another possibility, namely, that women low levels of life satisfaction that have experienced occupational injury are likely to have left work, and this may have biased the analysis.

According to previous studies, age is the most important risk factor of occupational injury [4]. The results the present study show a significant age-related increased risk of occupational injury in women, but not in men. In previous studies, shift work is known to increase the risk of occupational injury [24, 25]. However, the present study shows that occupational injury rates were 6.7 and 6.3% for shift workers and day-time workers, respectively, which was not a statistically significant difference. Furthermore, female shift workers had lower rates of occupational injury than day-time workers. It is possible these results were caused by differences in industry types, and that shift workers were engaged in industries with low risks of occupational injuries. Accordingly, we adjusted for industry classification to overcome this limitation.

Employment status and job tenure are indicators that are directly or indirectly linked to job proficiency. Although it was expected that longer job tenure and regular employment would reduce the risk of occupational injury, we found no statistical support for these expectations. We believe these negative results were because of sample bias. In the case of employment status, 89.6% of workers were in regular employment and 57.9% had job tenures exceeding 10 years. Because participants in this study were workers entitled to a health examination, temporary, part-time, and short job tenure workers were relatively poorly represented.

The mechanism by which satisfaction with life affects occupational injury is unknown. Previous studies have shown that psychological factors such as job stress [26] and depression [27] are associated with occupational injuries. On the other hand, satisfaction with life is known to be associated with psychological factors such as depression, stress, and anxiety [28]. Therefore, satisfaction with life is thought to be related to psychological factors that affect occupational injuries. Reduced satisfaction with life may increase the incidence of occupational injuries, by for example, reducing concentration at work and lowering participation rate in job safety education programs. In addition, reduced satisfaction with life may lead to decreases in physical abilities, such as, those associated with somatoform disorders, and induce disease, resulting in occupational injuries. On the other hand, workers exposed to factors that increase the risk of occupational injury, such as, an unsafe working environment, organizational problems, and excessive work, may have lower satisfaction with life. Thus, low satisfaction with life may not only increases the risk of occupational injuries directly, but also influences other factors that increase the risk of occupational injury.

The strength of this study is the prospective in design, which minimizes the misclassification of SWLS scores and occupational injury, and we controlled for potential confounding effects in the analyses. However, this study has some limitations. First, information on the occurrence of occupational injuries was obtained through self-reporting, which is well-known to be subject to recall bias. Nevertheless, a previous study showed good correlation between self-reported and recorded absences [29]. Second, the study subjects were limited to non-office workers who registered for health examination in a single large hospital; thus, findings may not apply to other workers. Third, there were several unmeasured confounders such as the availability of sick leave, income, and other psychosocial factors. Finally, SWLS scores were collected only in baseline survey, we were not able to account for satisfaction with life changes over time. There is a possibility that the association between SWLS and occupational injury may have been masked by the time elapsed between SWLS administration and occupational injury.

## Conclusion

This study indicates the risk of occupational injury is higher in workers not satisfied with life, and suggests paying more attention to satisfaction with life may promote worker health and minimize socioeconomic and productivity problems. Future studies should address not only life satisfaction but also the relationships between various psychological factors, such as, anxiety and depression and occupational injury.
